# Free Amino Acids and Volatile Aroma Compounds in Watermelon Rind, Flesh, and Three Rind-Flesh Juices

**DOI:** 10.3390/molecules27082536

**Published:** 2022-04-14

**Authors:** Xiaofen Du, Mindy Davila, Jessica Ramirez, Cierra Williams

**Affiliations:** Department of Nutrition and Food Sciences, Texas Woman’s University, Denton, TX 76204, USA; mdavila8@twu.edu (M.D.); jramirez37@twu.edu (J.R.); cwilliams59@twu.edu (C.W.)

**Keywords:** *Citrullus lanatus*, citrulline, rind, rind volatiles, watermelon juice, watermelon flavor

## Abstract

Watermelon rind is treated as agricultural waste, causing biomass loss and environmental issues. This study aimed to identify free amino acids and volatiles in watermelon rind, flesh, and rind-flesh juice blends with ratios of 10%, 20%, and 30%. Among the 16 free amino acids quantified, watermelon rind alone contained higher total amino acids (165 mg/100 g fresh weight) compared to flesh alone (146 mg/100 g). The rind had significantly higher (1.5×) and dominant amounts of citrulline and arginine (61.4 and 53.8 mg/100 g, respectively) than flesh. The rind, however, contained significantly lower amounts of essential amino acids. Volatile analysis showed that watermelon rind total volatiles (peak area) comprised only 15% of the flesh volatiles. Of the 126 volatiles identified, the rind alone contained 77 compounds; 56 of these presented in all five samples. Aldehydes and alcohols were most prevalent, accounting for >80% of the total volatiles in all samples. Nine-carbon aldehyde and alcohol compounds dominated both the flesh and rind, though the rind lacked the diversity of other aldehydes, alcohols, ketones, terpenes, terpenoids, esters and lactones that were more abundant in the watermelon flesh. Watermelon rind was characterized by the major aroma compounds above their thresholds, including 17 aldehydes and six unsaturated nine-carbon alcohols. This study demonstrated the potential for rind as a food or beverage supplement due to its key features such as concentrated citrulline and arginine, relatively low odor intensity, and valuable volatiles associated with fresh, green, cucumber-like aromas.

## 1. Introduction

Watermelon (*Citrullus lanatus*) is an essential and globally popular fruit; it constitutes 7% of the agricultural space dedicated to fruit and vegetable production [1]. In 2017, the global watermelon production was 118 million tonnes [2], with an estimated value of $60 billion. Watermelon fruit consists of peel/skin, rind (approximately 40% of total fruit weight), flesh (approximately 60% of total fruit weight), and seeds [2]. Despite being edible, the rind is often discarded before serving/processing, causing biomass loss [3]. The waste is currently sent to landfills, incinerated, used for animal feed, or composted, all of which are low value, costly, or have additional negative environmental effects [4]. The rind waste could be redirected to commercial use in the food industry considering its potential.

The potential value of watermelon rind has been recognized and explored. Approaches include converting rind polysaccharides into other products like bioethanol [3], biosorbent [5], and biochar [6]. Rind is also a food ingredient source for pectin [7,8] and citrulline [9,10,11]. Rind has been directly processed by pickling [12], as well as being incorporated into jams [13]. Watermelon rind in powder form has been applied in cakes [14,15], cookies [16,17], noodles [18,19], beef patties [20], and pork patties [21]. In addition, a few studies have investigated the use of whole watermelon rind as medium for microbials [22,23,24]. Overall, it was determined that incorporating rind into foods added value such as with pectin and specific amino acids.

In recent years, recycling valuable components from agro-food byproducts back into the food chain has gained great attention. Watermelon is a natural source of citrulline, a neutral non-essential α-amino acid and a precursor of L-arginine in the urea cycle [25]. Arginine is an essential amino acid which regulates blood flow and nitric oxide levels. Extensive studies have focused on citrulline in both watermelon flesh and rind [25,26]; however, fewer studies have investigated watermelon rind full amino acid profiles.

In addition to the mentioned food applications, the application of fresh rind in a food product has also been performed in our lab. Our previous consumer tests indicated that fresh rind’s sweet, sour taste and watermelon-like aroma is very different compared to the flesh [27]. Aroma, along with sweet (sugars) and sour (acids) tastes, is essential for fresh and processed fruit products. Flavor (aroma and taste) is gaining consideration as the major quality attribute. Although qualitative volatile characterization of watermelon flesh is well-documented [26,28], few studies have analyzed watermelon rind’s volatile compounds [29]. Quantitative volatile studies for rind and flesh are limited. Chemical and sensory analysis of the rind flavor profile would assist with the determination of the scope and suitable applications for rind usage in the food industry.

The present study aimed to address the potential application of watermelon rind in watermelon rind-flesh juices by evaluating the amino acid profiles and volatile aroma composition using instrumental analysis and quantification. Five samples were formulated for comparison: rind alone, flesh alone, and rind-flesh blends with rind portions of 10%, 20%, and 30%. The 20% rind sample was selected for this study based on the previously determined consumer acceptable level of 20% rind [27]. The 10% and 30% rind samples were included to monitor ratio changes which could potentially alter flavor perception.

## 2. Results

### 2.1. Free Amino Acids in Watermelon Rind, Flesh, and Rind-Flesh Blends

A total of 16 free amino acids were identified and quantified in the five watermelon samples. The identified amino acids included seven essential amino acids (Phe, Ile, Val, Leu, Met, His, and Lys), while His and Lys only had trace levels. Two other essential amino acids (Thr and Trp) were not detected. The remaining nine amino acids were Arg, Cit (citrulline), Ala, Ser, Pro, Gly, Asp, Asn, and Orn. Other non-essential amino acids such as Glu, Gln, Thr, Tyr, and Trp were also analyzed, but they were not detected in the five samples.

**Figure 1:**
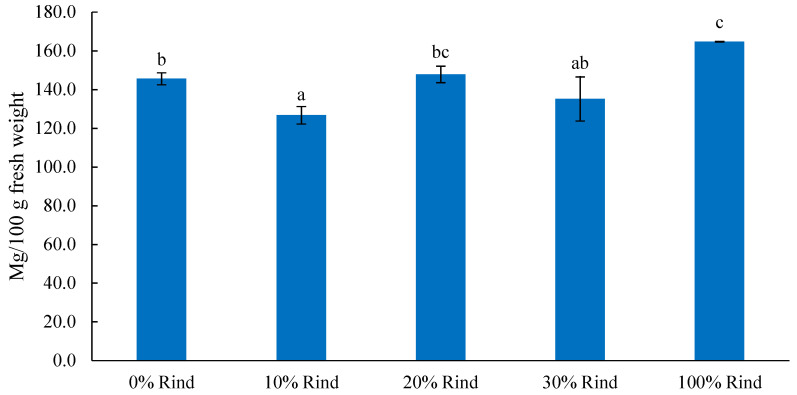
Total free amino acids (sum of 16 amino acids, mg/100 g fresh weight) in watermelon rind, flesh, and rind-flesh blends. Different letters (a–c) among five samples indicate significant differences according to one-way ANOVA and Tukey’s HSD test with *p* ≤ 0.05.

The sum of all amino acids in each sample ranged 127–165 mg/100 g fresh weight (0.13–0.17%), as shown in Figure 1. The rind alone contained the significantly (*p* ≤ 0.001) highest amount of total free amino acids (165 mg/100 g fresh weight), while flesh alone had 146 mg/100 g fresh weight.

The concentration of each free amino acid had a wide range in the five watermelon samples, as shown in Table 1. Cit had the highest concentration, ranging between 39.7 mg/100 g (0.04%) fresh weight in the 0% rind sample (entire flesh) to 61.4 mg/100 g (0.06%) fresh weight in 100% rind, approximately a 1.5-folddifference. The rind-flesh blends had Cit concentrations in between the samples with flesh only and rind only. A similar trend was observed for Arg. The 0% rind sample contained 36.7 mg/100 g fresh weight, while the 100% rind sample had 53.8 mg/100 g fresh weight, which was again found to be about a 1.5-fold difference.

Among the identified seven essential amino acids, Phe, Ile, Val, and Leu had concentrations above 1.0 mg/100 g fresh weight (Table 1). The 0% rind sample (flesh only) contained the highest amount. As rind increased in the samples, these essential amino acids decreased; the lowest concentrations were in the 100% rind sample. The flesh only sample had about 4.1 times higher total essential amino acids than the rind only sample.

Additionally, five other amino acids with concentrations higher than 1.0 mg/100 g fresh weight were Ala, Ser, Pro, Asp, and Asn (Table 1). The concentration for Ser and Asp increased along with the rind percentage in the samples; 100% rind had 2.4–3.5 times the content found in flesh alone. The concentrations for Ala, Pro, and Asn decreased when rind potency increased; the difference between only rind and only flesh samples was 1.2–2.1 times.

Amino acids are not only associated with nutritional value, but also have the potential to contribute bitter, sweet, and umami tastes, although some are tasteless. The most dominant amino acids in the five samples, Arg and Cit, are associated with bitterness taste. However, their concentrations were below their perception thresholds, for example, 435.5 mg/100 g for Arg in water [30]. The other seven quantified essential amino acids (Phe, Ile, Val, Leu, Met, His, and Lys) have a potential bitter taste, but their concentrations were also all below their thresholds. The concentrations for the four sweet associated amino acids (Ala, Ser, Pro, and Gly) were similarly below their thresholds. Lastly, Asp (umami-related) did not surpass its 53 mg/100 g in water threshold, either [30].

### 2.2. Watermelon Rind, Flesh, and Rind-Flesh Blend Brix and TA

**Figure 2:**
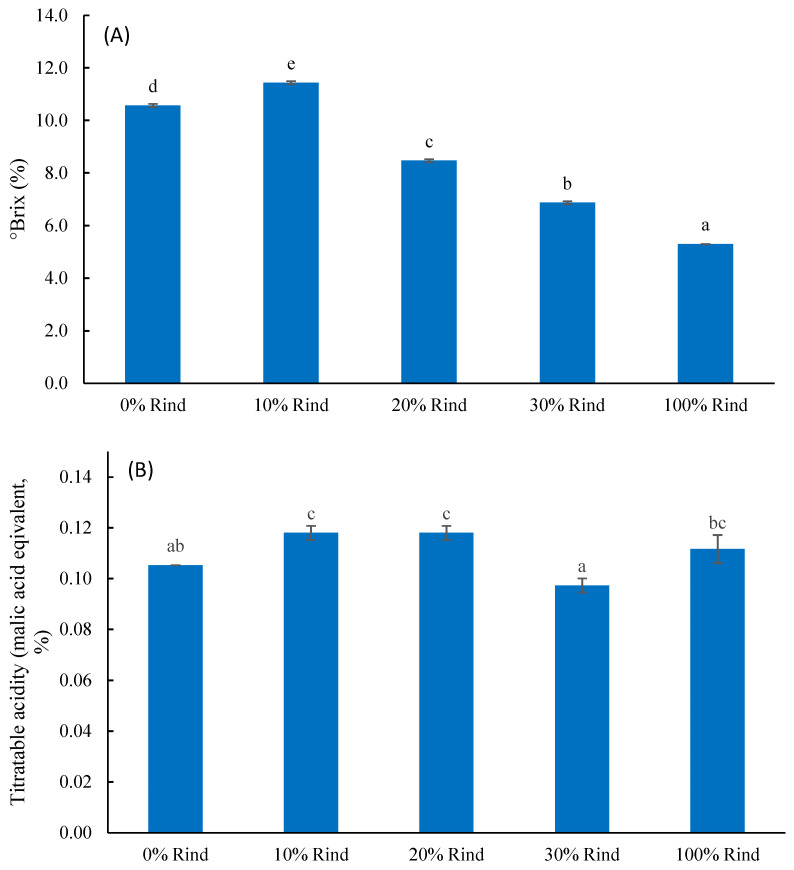
Mean values of °Brix (**A**) and titratable acidity (**B**) (TA, reported in % malic acid equivalents) in watermelon rind, flesh, and rind-flesh blends. Different letters (a–d) among five samples indicate significant differences according to one-way ANOVA and Tukey’s HSD test with *p* ≤ 0.05.

Measured Brix values represent the total soluble solid content, which mainly includes sugars, amino acids, organic acids, soluble pectin, phenolic compounds, and minerals in watermelon juice [26]. This study showed that °Brix in the sample flesh alone was 10.6, and the °Brix significantly (*p* ≤ 0.05) dropped to 5.3 for 100% rind (Figure 2A). The blends had °Brix in between the values for pure flesh and rind. This suggests that rind should be significantly less sweet compared to flesh.

Watermelon acidity could be measured by titratable acidity (TA, the total acid concentration). This study showed the TA, reported in malic acid equivalents, ranged 0.10–0.12% in the five samples (Figure 2B). Although the range was small, it showed a significant difference among the samples.

### 2.3. Watermelon Rind, Flesh, and Rind-Flesh Blend Volatiles—An Overview

**Figure 3:**
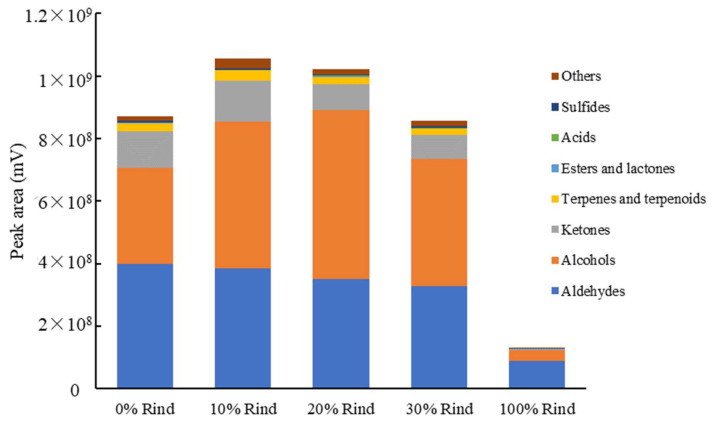
Abundance (peak area, mV) of grouped volatiles in watermelon rind, flesh, and rind-flesh blends using SPME-GC-MS analysis.

A total of 126 volatile compounds with a range of 77–114 unique compounds were identified in the five samples. Based on their functional groups, the identified volatiles were classified into eight groups: 32 aldehydes, 32 alcohols, 18 ketones, 20 terpenes and terpenoids, nine esters and lactones, five acids, three sulfurs, and seven others. As shown in Figure 3, the most abundant volatiles overall were aldehydes, accounting for 34.2–68.9% of the total volatiles in the samples, followed by alcohols (24.9–53.2%) and ketones (2.7–13.6%). The remaining five groups of volatile compounds only accounted for 3.5–6.8% of the total volatiles.

The total volatile content (a sum of all eight group peak areas) slightly increased along with the rind percentage in the 10% and 20% rind-flesh blends; the 30% rind blend was similar to the flesh only sample. However, Figure 3 demonstrates watermelon rind alone contained a markedly smaller volatile content, which was approximately 15% of the flesh (0% rind), 15% of the blend containing the most rind (30% rind blend), and 12% of the other two blends (10% and 20% rind). Since the total volatile content was so heterogeneous, the ratio of each group of volatiles also changed accordingly. For example, the total peak area for aldehydes in the 100% rind sample was smallest compared to the other four samples; however, the 100% rind sample contained the highest ratio of aldehydes, 68.9% vs. 34.2–45.9% for the other four samples. Conversely, the 100% rind sample contained the lowest ratio of alcohols (24.9% in the 0% rind sample compared to 35.1–53.2%) and ketones (2.7% in the 0% rind sample compared to 7.8–13.6%).

### 2.4. Common Volatiles in Watermelon Rind, Flesh, and Rind-Flesh Blends

The distribution of the volatile groups among the rind-flesh blends are illustrated by supplemental data (see Supplementary Materials Table S1). There were 112 volatiles found in the 0% rind sample compared to 77 found in the 100% rind sample. The variety of volatiles decreased slightly with increasing rind content; 114, 113, and 110 compounds were found in the 10%, 20%, and 30% rind samples, respectively. There were around 61 common volatile compounds (out of a total of 126 compounds) recovered from all five samples (those with and without rind). These common volatiles were 16 aldehydes, 19 alcohols, six ketones, eight terpenes and terpenoids, two esters and lactones, three acids, two sulfurs, and five others. Aldehydes and alcohols were the most common volatiles in all samples.

The 11 most dominant volatile compounds (at least 1% peak area) were acetaldehyde (3.9–17.0%), hexanal (6.9–14.2%), nonanal (1.8–3.7%), (*Z*)-6-nonenal (1.6–3.6%), (*E*)-2-nonenal (8.1–10.6%), (*E*,*Z*)-2,6-nonadienal (4.6–11.7%), hexanol (3.1–4.4%), (*Z*)-3-nonen-1-ol (3.2–19.6%), (*Z*,*Z*)-3,6-nonadien-1-ol (1.9–11.8%), and 6-methyl-5-hepten-2-one (2.2–12.4%). Although most abundant, these volatiles did not distribute evenly in the five samples. Volatiles present in lesser abundance in the 100% rind sample but significantly (*p* < 0.001) rich in flesh were nonanal, (*Z*)-6-nonenal, (*Z*)-3-nonen-1-ol, (*Z*,*Z*)-3,6-nonadien-1-ol, and 6-methyl-5-hepten-2-one. The green, fatty, and fresh volatiles acetaldehyde, hexanal, (*E*)-2-nonenal, and (*E*,*Z*)-2,6-nonadienal were interestingly more abundant in the 100% rind sample compared to the 0% rind sample, *p* < 0.001. In addition to those most abundant common volatiles, the remaining 50 volatiles common to all five samples had peak areas less than 1%, which included 11 aldehydes, 15 alcohols, four ketones, and seven terpenes and terpenoids (Table S1).

### 2.5. Different Volatiles in Watermelon Rind and Flesh

Except those 61 common volatiles identified from all samples, 65 volatiles were not universally present (Table S1). There were 46 volatile compounds only in samples containing at least some flesh, which were seven aldehydes, 10 alcohols, 12 ketones, 10 terpenes and terpenoids, four esters and lactones, and two acids.

Ketones, terpenes and terpenoids, and esters and lactones were the volatiles mainly lacking in rind. The most abundant of those included heptanal (0.13–0.22%), octanal (0.18–0.31%), (*E*)-2-heptenal (0.42%, only recovered from the 0% rind sample), methanol (0.45–0.85%), 6-methylhept-5-en-2-ol (0.87–1.41%), (*E*)-2-octen-1-ol (0.10–0.40%), 3-ethyl-3-undecanol (0.47–0.55%), 3-octanone (0.14–0.30%), (*E*,*Z*)-3,5-octadien-2-one (0.05–0.25%), and hexanoic acid (0.10–0.15%), which were more abundant in the samples with less rind, 0% and 10% rind samples, compared to the 20% and 30% rind samples, *p* ≤ 0.05. The remaining compounds in the samples containing watermelon flesh had peak area less than 0.1%.

There were 13 volatiles that were identified only from samples which contained rind (100%), including butanal (0.09–1.19%), pentanal (0.20–1.57%), decanal (0.02–0.07%), benzaldehyde (0.05–0.52%), (*Z*)-2-penten-1-ol (0.22–0.39%), tetrahydrolinalool (0.01–0.07%), isobornyl acetate (0.01–0.10%), methyl (*Z*,*Z*)-9,12-octadecadienoate (0.02%), and dimethyl trisulfide (0.01–0.17%). All of those volatiles increased in abundance with increasing rind except for (*Z*)-2-penten-1-ol and decanal, which decreased (*p* ≤ 0.05). Isobornyl acetate and tetrahydrolinalool have never been reported in watermelon. Volatiles that were exclusively present in the 100% rind sample were 2-methyl-3-methylene-cyclopentane carboxaldehyde (0.27%) and naphthalene (0.22%).

### 2.6. Differentiation of Five Sample with Heat Map

**Figure 4:**
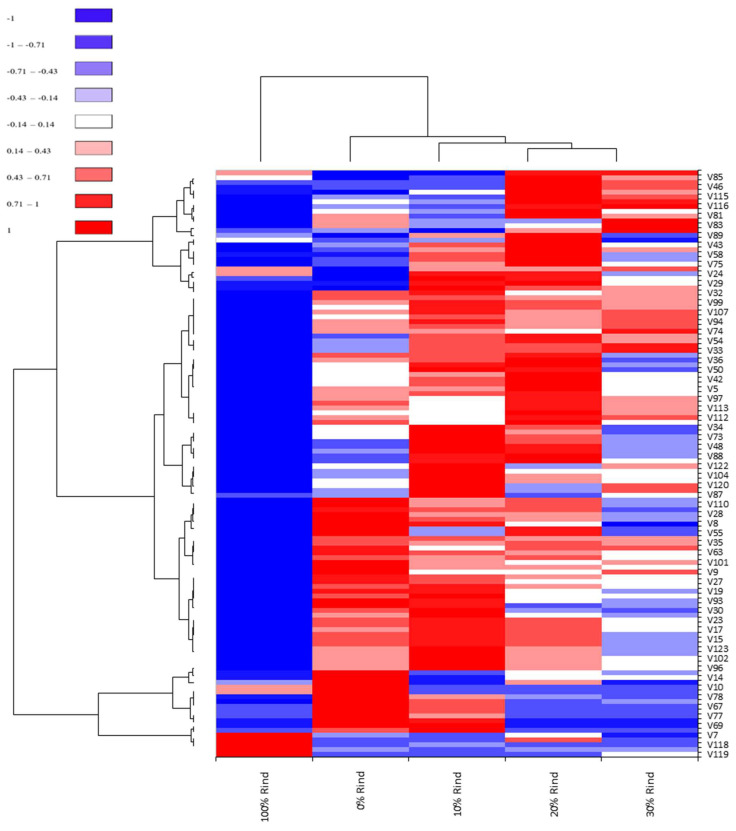
Heat map of 61 selected volatiles (peak area, filtration interquartile range threshold = 0.01) in watermelon rind, flesh, and rind-flesh blends. V: volatiles, which could be found in Table S1.

To visualize and compare individual volatiles in the four rind-flesh blend samples and 100% rind sample, a heat map cluster analysis was conducted (Figure 4). The four blends were clustered according to their flesh content in the blends, which were separate from the 100% rind sample. The 100% rind sample showed negative correlations to almost all selected volatiles (blue color in Figure 4), but showed positive correlations to volatile compounds such as dimethyl disulfide, dimethyl trisulfide, (*E*)-2-butenal, (*E*)-2-pentenal, benzaldehyde, and (*Z*)-6-octen-2-one. An Analysis further clustered the rind-flesh blends by separating the 0% and 10% rind samples and grouping together the 20% and 30% rind samples. The 20% and 30% rind samples were grouped together based on their shared volatiles derived from rind and other shared volatiles derived from flesh (both blue and red colors in Figure 4). Compared to the 20% and 30% rind samples, the 0% and 10% rind samples were more positively correlated (red color in Figure 4) with volatiles derived from flesh like (*Z*)-6-nonenal, (*Z*)-2-nonenal, 4-oxononanal, (*E*)-2-octenal, (*Z*)-citral, (*E*)-geranylacetone, and 6-methyl-5-hepten-2-one. The heat map clearly differentiates samples based on their volatile content from rind and flesh.

### 2.7. Selected Volatile Compound Quantification

Since aldehydes and alcohols were the most abundant volatiles in watermelon rind, these volatiles were selected for quantification to investigate the aroma-active volatiles in the rind with the odor activity values (OAVs). A total of 27 compounds were quantified, which accounted for approximately 80% of the total peak area for each sample. These compounds included 17 aldehydes, nine alcohols, and one ketone, or they also included six six-carbon and 14 nine-carbon compounds (Table 2). Results indicated nine-carbon aldehydes and alcohols were the most dominant volatiles in flesh, and aldehydes were most dominant in watermelon rind. The concentration of these 27 compounds in watermelon rind (100% rind sample) ranged from 0.7–2349 µg/kg fresh weight, while in watermelon flesh (0% rind sample) it ranged from 0–4255 µg/kg fresh weight.

The major aroma characteristics for the 27 quantified volatiles were fresh, green, cucumber-like, and fruity with fatty nuances (Table 2). According to their corresponding concentrations and thresholds, volatile compounds potentially contributing aromas to watermelon rind included five unsaturated nine-carbon alcohols (*Z*-3-nonen-1-ol; *Z*-6-nonen-1-ol; *E*,*Z*-3,6-nonadien-1-ol; *Z*,*Z*-3,6-nonadien-1-ol; and *E*,*Z*-2,6-Nonadien-1-ol) and 16 aldehydes. The 16 aldehydes consisted of three six-carbon aldehydes (hexanal, *Z*-3-hexenal, and *E*-2-hexenal), all eight nine-carbon aldehydes (nonanal; *E*-6-nonenal; *Z*-6-nonenal; *Z*-2-nonenal; *E*-2-nonenal; *E*,*E*-2,6-nonadienal; and *E*,*Z*-2,6-nonadienal), and six other compounds (acetaldehyde, propanal, 2-methylbutanal, 3-methylbtuanal, pentanal, and *E*-2-octenal).

## 3. Discussion

Watermelon is a rich source of amino acids and other nutrients [2]. Our current study indicated that watermelon rind had higher total free amino acids than flesh; however, the free amino acids in watermelon rind had less diversity. Cit and Arg were the two most abundant free amino acids in both watermelon rind and flesh, and the concentration of Cit significantly increased along with rind concentration in the samples, with the rind alone (100% rind) containing the highest amount of Cit. The finding of higher Cit content in rind than flesh was consistent with literature reports [9,25], though there are also conflicting reports [10]. The concentration of Cit varies a lot in literature, which depends on genotype, environmental factors, and analytical methods [25]. Similar to Cit and in accordance with the literature, the concentration of Arg increased along with rind in the samples in this study [25]. The results indicated the potential nutritional value for watermelon rind.

Out of nine essential amino acids, seven were identified in watermelon flesh and rind in this study. Rind had a lower content of all essential amino acids, indicating lower nutritional quality compared to flesh. In addition to nutritional value, amino acids have the potential to contribute to tastes such as bitter, sweet, and umami. However, our results indicated that all measured 16 free amino acids had concentrations below their perception thresholds; this suggests that free amino acids in watermelon most likely do not contribute flavor/taste. The negligible taste contribution of watermelon amino acids might explain the absence of this subject in literature.

The most critical sensory quality trait of watermelon is sweetness, which relies mainly on mono- and di-saccharides in addition to other soluble solids [26]. The sugar content in watermelon usually reflects the fruit quality and sweetness. This study showed that watermelon rind had significantly less °Brix compared to flesh, implying that rind should have a significantly less sweet taste compared to flesh. The results were consistent with our previous consumer study that demonstrated that rind incorporation diminished the sweet taste but increased the sourness taste of watermelon juice [27]. A few studies have reported °Brix in watermelon flesh, which has an average value around 10 [31,32]. To the best of our knowledge, °Brix or total soluble solid in watermelon rind has been never been reported. Organic acids, such as malic, citric, and oxalic acids contribute to the sourness taste, but the concentration in watermelon flesh is generally low, <0.5% [33]. Organic acids in watermelon rind have not been reported in the literature. °Brix and TA for the samples in this study were consistent with these literature reports. Commonly, °Brix and TA in plant fruits vary significantly with endogenous factors (genotype, maturity stage, and fruit sampling area) and environmental factors.

Aside from the sweet taste, watermelon aroma is the next most important contributor to sensory quality. Approximately 300 volatiles have been identified in watermelon flesh so far [34]; influential factors include watermelon genotype, agro-environmental condition, and the volatile analysis method. Hence, the number of watermelon volatiles obtained by each researcher varies substantially [35,36,37,38,39,40,41]. Their works show the identification of a lesser number of watermelon compounds (41–71) than those found in this study (126 total in rind, flesh, and rind-flesh blends, Table S1).

Of the eight grouped volatiles in this study, the common conclusion was that aldehydes, alcohols, and ketones were the most dominant volatiles in the five samples. The results were consistent with findings in the literature that summed aldehydes and alcohols in watermelon flesh, especially nine-carbon linear compounds, can account for up to 77% of the total volatiles [28,36,42]. Although the trend for dominant volatiles was similar for flesh and rind, the abundance of the total volatiles varied tremendously between the samples with flesh and without flesh (100% rind). The 10% rind sample had the overall greatest abundance (peak area) of volatiles, followed by 20% rind, 0% rind, and 30% rind samples. It was hard to explain why the total volatile abundance in 10% rind and 20% rind samples was higher than the 0% rind sample. The phenomenon might be caused by the impact of matrix (ratio of flesh and rind causing different volatile binding and release) on SPME fiber absorption. Nevertheless, the 100% rind sample only had around 15% of the total volatiles in the flesh sample. The low volatile abundance in watermelon rind would be easy to mask in food products where a melon flavor is not desired, meanwhile adding nutritional value such as Arg and Cit.

A detailed list of volatile compounds in watermelon rind is reported by our most recent publication [29], which results in half of the identified volatiles commonly presenting in both rind and flesh. The present study’s results also revealed that the rind of watermelon possessed some aromas identical to the flesh, though volatiles responsible for most melon-like aromas were generally less abundant in the 100% rind sample compared to the 0% rind sample. The prevalence of nine-carbon volatile compounds in the tested samples observed in this study, including (*E*)-2-nonenal; (*Z*)-3-nonen-1-ol; (*E*,*Z*)-2,6-nonadienal; (*Z*)-6-nonenal; nonanal; and (*Z*,*Z*)-3,6-nonadien-1-ol, have been described to have melon, fresh, sweet, fruity, floral, cucumber, and green related aromas [35,43]. The finding of flavor compounds in watermelon rind that were identical to those in flesh affirmed the potential of rind as a melon flavor enhancer. The use of rind as a flavor enhancer was tested in raw watermelon rind-flesh blends by consumers in an earlier study [27]. The volatiles in those blends were identified to uncover the interactions between the chemical and sensorial aspects of rind.

Despite the presence of those recognizable volatiles, watermelon rind consumption is not as popular as is the flesh, suggesting that there are key differences between their flavor profiles. This difference was reflected in not only the total number of identified volatiles, but also their abundance (peak area). Rind concentration in the samples shared a direct relationship with the abundance of nine-carbon alcohols and nine-carbon aldehydes, but the rind lacked the diverse aldehydes, alcohols, terpenoids, and ketones characterizing the flesh. The heat map further accentuated the volatile profile differences between rind and flesh by coloring the rind mostly blue (negative correlation to most identified volatiles) and separating it from the other samples. In other words, samples with greater amounts of rind retained classic melon flavors, but they were missing additional flavor nuances (fresh, green, fatty, citrus, floral, creamy, etc.) contributed by the unique volatile compounds in the flesh.

In addition to the knowledge of shared and distinctive volatiles distributed in watermelon flesh and rind, it would be ideal to know the aroma-contributors in watermelon rind–an unexplored topic. Extensive studies have been conducted to investigate aroma-active compounds in watermelon flesh (juice) by directly using GC-olfactometry (GC-O) as well as odor activity value (OAV) computation [42,44,45]. OAVs are the ratio between the quantified volatile content and the corresponding odor threshold level. The majority of studies name nine-carbon aldehydes and alcohols as aroma contributors. Our current study found aldehydes (low volatility, six-carbon and nine-carbon) and unsaturated nine-carbon alcohols to be the major contributors for watermelon rind aroma, suggesting that watermelon rind smelled fresh, green, grassy, and cucumber-like, with intensity much lower than watermelon flesh.

Although the dominant aroma compounds were nine-carbon aldehydes and alcohols, it should be pointed out that watermelon flesh should have a richer aroma profile than the description in the literature. It has been mentioned that the combination of nine-carbon alcohols and aldehydes was found to be insufficient to reconstruct the intricate aroma of watermelon [46]. Watermelon flesh (juice) has been described to have fresh, green, cucumber-like, melon-like, fruity, and floral notes [47]. The fruity and floral notes can most likely be attributed to other volatiles groups (not aldehydes or alcohols). For example, certain terpenoids (β-ionone) and esters (hexyl acetate and γ-nonalactone) might contribute floral, fruity, and sweet notes. However, in watermelon rind, these volatiles were lacking.

Volatile biosynthesis in watermelon flesh mainly involves the LOX (lipoxygenase) pathway, terpenoid pathway, and degradation and biosynthesis of amino acids, which require precursors of fatty acids, carotenoids, and amino acids, respectively [28]. The LOX metabolite pathway is the most dominant in watermelon and, therefore, is very well studied; it generates volatiles such as aldehydes, alcohols, ketones, esters, and acids. The dominance of these volatiles found by the current study confirmed that LOX is the most active biological pathway in watermelon flesh and rind. The second most important metabolite pathway is the terpenoid pathway, which generates terpenes, terpenoids, and norisoprenoids; common carotenoids involved in this pathway include lycopene and β-carotene. For example, the major norisoprenoid volatiles present in lycopene-containing watermelons are geranial, neral, 6-methyl-5-hepten-2-one, 2,6-dimethylhept-5-1-al, 2,3-epoxygeranial, (*E*,*E*)-pseudoionone, geranyl acetone, and farnesyl acetone [48]. Compounds derived from β-carotene include β-ionone, dihydroactinodiolide, and β-cyclocitral [48]. In this study, watermelon flesh had diverse terpenes, terpenoids, and norisoprenoids, which were lacking in watermelon rind. This might be associated with the lack of carotenoids (generally yellow, red or orange in color) in watermelon rind, as the watermelon rind had a white-green color.

## 4. Materials and Methods

### 4.1. Chemicals

An EZ:faast^TM^ amino acid analysis kit (category number KG0-7166) was purchased from Phenomenex (Torrance, CA, USA). The amino acid kit includes two sets of amino acid standards which were SD1 (23 amino acids) and SD2 (3 amino acids). Two other amino acids, citrulline and arginine, were analytical standard grade and obtained from MilliporeSigma (Milwaukee, WI, USA). Water, methanol, ethanol, and acetonitrile were HPLC grade and obtained from Fisher Scientific (Fair Lawn, NJ, USA). Phosphoric acid and monopotassium phosphate were analytical grade and obtained from Fisher Scientific. A total of 33 volatile standards (acetaldehyde; pentanal; hexanal; pentyl acetate; 2-heptanone; (*E*)-2-hexenal; octanal; 6-methyl-5-heptanone; hexanol; *cis*-3-hexenol; nonanal; *trans*-2-hexenol; *trans*-2-octenal; *cis*-6-nonenal; benzaldehyde; *trans*-2-nonenal; linalool; 2,6-nonadienal; *E*-2-decanal; nonanol; *cis*-6-nonenol; *E*-2-nonenol; 2,6-nonadienol; *E*,*E*-2,4-decadienal; tridecanal; geraniol; hexanoic acid; benzyl alcohol; cinnamaldehyde; β-ionone; γ-nonalactone; ethyl cinnamate; and nonanoic acid) with purity ≥ 98% were purchased from MilliporeSigma. Sodium chloride was purchased from Fisher (ACROS Organics, Fair Lawn, NJ, USA). The C6–C26 alkane mixture was obtained from MilliporeSigma.

### 4.2. Watermelon Rind-Flesh Blend Samples

Five samples (100% flesh; rind-flesh blends at ratios of 10:90, 20:80, and 30:70 *w*/*w*; and 100% rind) were used for this study. Sample information was reported in our previous consumer study [27]. The five samples were subjected to free amino acid, °Brix, TA, and volatile profile analysis and quantification in this study. Briefly, the samples were prepared with Captivation and Exclamation melon cultivars, which were grown at the Texas A&M AgriLife Research & Extension Center in Lubbock, TX, USA. The fresh rinds (green/white colored outer edge portion of the fruit, approximately 1 cm thick) from these two watermelons were completely cleaned, with elimination of both the internal part (the endocarp or flesh) and the streaked green external skin/peel. The watermelon flesh of each cultivar was diced and mixed thoroughly to ensure homogeneity of the two cultivars. Separately, all melon rinds were diced and mixed well. Rind-flesh juice blends were prepared by blending 0%, 10%, 20%, 30%, and 100% (*w*/*w*) rind in flesh mixtures until a homogeneous consistency was reached (20–40 s). Samples were immediately frozen (−20 °C) until chemical analysis. On the day of analysis, samples were thawed at room temperature to a liquid state.

### 4.3. Free Amino Acid Quantification Using GC-MS

First, each sample was thawed and 4.0 g was randomly portioned and combined with 40 mL DI water. The mixture was centrifuged at 4000 rpm for 10 min. A 100 µL sample of each supernatant was subsequently derivatized per the EZ:faast Free (Physiological) Amino Acid Analysis by GC-MS kit instructions.

Gas chromatography-mass spectrometry (GC-MS) analysis of the derivatized samples was performed using equipment specifications listed in our previous publication [49]. Each amino acid was identified by matching the kit’s provided major ions and retention times to the sample ion spectra chromatogram. Identification was confirmed using the kit’s SD1 and SD2 amino acid standards.

A total of 14 free amino acids were quantified using the constructed calibration curves with the EZ:faast–GC-MS analysis kit [49]. Briefly, 13 aliquots (equivalent to 12.5–3200 nmol/mL) of standard reagents SD1 and SD2 were analyzed via EZ:faast–GC-MS in the same manner as the samples. The calibration curves had 6–13 levels (r^2^ ≥ 0.99 excluding serine r^2^ = 0.97).

### 4.4. Thermal Unstable Amino Acid Quantification Using HPLC-UV

Two thermally unstable amino acids, arginine and citrulline, were quantified using high performance liquid chromatography (HPLC) with ultraviolet (UV) detection. Thawed watermelon samples were centrifuged (4000 rpm, 10 min) and 1 mL of the supernatant was combined with 9 mL of DI water (10× dilution). The diluted samples were then passed through a 0.45 µm syringe prior to HPLC injection.

A Restek Ultra AQ C18 column (5 µm, 250 × 4.6 mm) was connected to a HPLC system (Shimadzu USA Manufacturing, Canby, OR, USA) with the following configuration: a binary pump (LC-20AR), a degassing unit (DGU-20A 3R), an autosampler with a 100 µL sample loop (SIL-20A), and an UV detector (SPD-20A). Gradient elution utilized the following 2 solutions: mobile phase A (0.1% orthophosphoric acid [stock-85% purity] in water, *v*/*v*) and mobile phase B (100% acetonitrile). Injection volume and flow rate were 20 μL and 1.0 mL/min, respectively. The UV detection wavelength was 200 nm. Mobile phase A began at 100%, reaching 5% by 15 min retention time. By 20 min retention time, mobile phase A was back at 100%. The column was flushed with 95% acetonitrile at the end of each chromatographic run, followed by 10 min at the initial solvent composition. Total analysis time was 35 min.

A 0.64 mg/mL DI water stock solution was prepared separately for citrulline and arginine, and then serially diluted as follows: 0.64, 0.32, 0.16, 0.08, 0.04, 0.02, and 0.01 mg/mL. These 14 solutions were run in the same manner as the samples. Peak areas were used to construct linear calibration curves, which were not forced through the origin. A range of five to seven calibration levels were employed in order to obtain sufficient quantification ranges (citrulline: 0.01–0.64 mg/mL, arginine: 0.02–0.32 mg/mL) with r^2^ ≥ 0.99.

### 4.5. °Brix and TA Measurement

As indicated in our previous publication [27], °Brix was measured in the thawed watermelon samples with a pocket refractometer (Atago Co., Ltd., Tokyo, Japan). Titratable acidity (TA, 1:12 dilution) was measured using a Titrando (Metrohm 888 Titrando, Herisau, Switzerland) and expressed as % malic acid equivalents. All dilutions were performed using DI water.

### 4.6. Volatile Analysis Using SPME-GC-MS

Volatile compounds in thawed watermelon samples were analyzed using solid-phase microextraction–gas chromatography (SPME–GC-MS) as described in our previous publication [29]. To begin, 3 g of each sample was added into 20 mL glass headspace vials (prefilled with 1 g of NaCl). The vials were sealed with 18 mm magnetic screw craps with a PTFE blue silicone septum. The sample vial was incubated for 15 min at 40 °C to reach equilibrium. After equilibration, a SPME fiber was injected into the vial headspace for volatile extraction for 20 min, with agitation at 250 rpm. The SPME fiber was coated with divinylbenzene/carboxen/polydimethylsiloxane (DVB/CAR/PDMS, 50/30 μm film thickness; Supelco, Bellefonte, PA, USA). An automated AOC-6000 sampler (Shimadzu, Columbia, MD, USA) was used for sample vial incubation, headspace volatile equilibration, and volatile extraction with SPME fiber.

The SPME fiber was desorbed for 3 min at the splitless injection port (250 °C) of the GC unit (GC-2010 Plus coupled with a QP2020 mass spectrometer; Shimadzu, Columbia, MD, USA). A helium carrier gas flowed at 1 mL/min. The oven temperature was held for 1 min at 40 °C, ramped up at 5 °C/min to 230 °C, and held for 5 min at 230 °C. A 30.0 m, 0.25 mm i.d., 0.25 µm film thickness ZB-Wax column (Phenomenex, Torrance, CA, USA) was used to separate compounds. The mass spectrometer used an ionization source at 200 °C, an interface temperature of 230 °C, and a fragment range of 35 to 350 *m/z* [29].

To calculate the linear retention index (LRI), alkane standards were analyzed on the SPME-GC-MS. A straight-chain, C6-C26 alkane mixture was prepared by adding the alkane stock solution to pentane in a 1:100 ratio. SPME parameters included a 1 min equilibration time and 5 min extraction time. GC-MS parameters were identical to those used for the watermelon samples. The linear retention indices of the identified watermelon blend volatile compounds were calculated.

Compounds were identified by matching their ion spectra chromatogram with those from the National Institute of Standards and Technology (NIST) library. Identity confirmation was completed by comparing their LRI with values from the webbook.nist.gov database (accessed on 5 April 2022). Relative compound quantities (percent of peak area) were calculated.

### 4.7. Volatile Quantification with Calibration Curves

Twenty-seven key volatiles were selected for quantification in the watermelon samples, based on our previous study [29]. To do so, calibration curves for each compound were created and applied to sample chromatograms for final concentration calculation.

To make the curves, deodorized rind samples were analyzed with SPME-GC-MS before and after spiking with selected volatile standards. To deodorize, approximately 100 g of thawed watermelon sample was rotatory evaporated at 45 °C for 2 h. The lost volume was compensated with DI water. The deodorized watermelon rind samples were then analyzed via SPME-GC-MS using the same conditions as for the original five samples, and the chromatogram for this deodorized watermelon rind was treated as a blank. The deodorized rind samples were subsequently spiked with seven known concentration levels of a mixture of 27 standard compounds, and likewise analyzed via SPME-GC-MS. The peak areas for standard addition (corrected with blanks) were used to construct calibration curves. All calibration curves were linear with r^2^ ≥ 0.98.

### 4.8. Statistical Analysis

Each analysis method, namely free amino acid, °Brix, TA, and volatile (quantitative and qualitative peak area), was conducted in triplicate. One-way ANOVA and Tukey’s HSD test were performed on all data collected in this study. Heat map cluster analysis was used to visualize patterns in volatile abundances across samples. Volatiles with an interquartile range below 0.01 were removed from heat map analysis due to their low variability and to improve the clarity of the heat map. ANOVA utilized SPSS version 25 (IBM SPSS, Armonk, NY, USA), while the heat map cluster analysis used XLSTAT version 2019.4.1 (Addinsoft, New York, NY, USA). All tests assumed a significance level of *p* ≤ 0.05.

## 5. Conclusions

Free amino acid profiles and volatile aroma compounds in watermelon rind were analyzed in this study. Compared to watermelon flesh, watermelon rind contained a higher amount of total amino acids due to its concentrated Cit and Arg content; however, the rind had a lower amount of essential amino acids. The results suggest that watermelon rind could be used to nutritionally supplement food and beverages with Arg and Cit. Watermelon rind had a much lower °Brix and, consequently, a less sweet taste. Watermelon rind also contained a much lower amount of volatiles, only 15% compared to the flesh. The relatively “bland” flavor (taste and aroma) for watermelon rind might have less interference when it is used as an ingredient in processed foods. Also, fewer unique volatile compounds were recovered from watermelon rind compared to flesh. Both watermelon rind and flesh, however, were predominant with nine-carbon aldehydes and alcohols, which were potentially associated with fresh, green, and cucumber-like aromas. These volatiles in the rind might enhance aroma for the rind used in food products. The study provides knowledge that watermelon rind could be a promising supplemental ingredient for food and beverages. Manufacturers of food products can apply this research to develop products using watermelon rind waste for its nutritional value and aroma characteristics.

## Figures and Tables

**Table 1:** Mean values (mg/100 g fresh weight) of free amino acids identified and quantified in watermelon rind, flesh, and rind-flesh blends.

Type	Amino Acids	0% Rind	10% Rind	20% Rind	30% Rind	100% Rind	*p*-Values
E, bitter	Phe (Phenylalanine)	14.23 d	8.93 b	11.46 c	8.26 b	2.69 a	<0.001
E, bitter	Ile (Isoleucine)	10.79 d	7.62 b	8.95 c	6.99 b	2.37 a	<0.001
E, bitter	Val (Valine)	8.78 d	6.39 c	6.61 c	5.39 b	3.24 a	<0.001
E, bitter	Leu (Leucine)	2.37 c	1.86 bc	1.95 bc	1.59 b	0.61 a	<0.001
E, bitter	Met (Methionine)	0.63 b	0.79 bc	1.00 c	0.98 c	nd a	<0.001
E, bitter	His (Histidine)	nd a	nd a	nd a	nd a	tr a	0.190
E, bitter	Lys (Lysine)	nd a	nd a	nd a	tr a	tr a	0.390
Bitter	Arg (Arginine)	36.73 a	38.13 b	38.47 b	39.30 c	53.77 d	<0.001
Unknown	Cit (Citrulline)	39.73 a	41.30 a	44.43 b	45.73 b	61.43 c	<0.001
Sweet	Ala (Alanine)	14.42 d	9.05 b	12.13 c	9.06 b	6.78 a	<0.001
Sweet	Ser (Serine)	7.36 a	8.71 a	12.23 b	12.40 b	18.02 c	<0.001
Sweet	Pro (Proline)	6.07 d	3.58 a	5.08 c	3.94 b	3.31 a	<0.001
Sweet	Gly (Glycine)	tr a	nd a	nd a	0.27 a	0.37 a	0.042
Umami	Asp (Aspartic acid)	2.02 a	1.59 a	3.64 b	3.40 b	7.09 c	<0.001
Tasteless	Asn (Asparagine)	3.18 cd	2.36 a	3.32 d	2.81 bc	2.71 ab	0.001
Tasteless	Orn (Ornithine)	0.26 a	0.21 a	0.16 a	0.43 a	1.71 b	0.001

Different letters (a–d) within each row indicate significant differences of each amino acids among five samples according to one-way ANOVA and Tukey’s HSD test (α ≤ 0.05). E: essential amino acids; nd: not detected; tr: trace level, indicating two instances of no detection and one instance of small detection within the triplicate.

**Table 2:** Volatile compounds in watermelon rind-flesh blends (µg/kg).

LRI	Compound	0% Rind	10% Rind	20% Rind	30% Rind	100% Rind	OT (µg/kg)	Odor Descriptions
672	Acetaldehyde	4255.2 b	4546.8 b	4881.2 b	4117.3 b	2348.7 a	15	Fresh, green, fruity
794	Propanal	84.9 c	80.9 b	79.5 b	80.7 bc	1.4 a	10	Musty, earthy, vegetative
882	2-Methylbutanal	34.0 c	30.2 b	33.3 c	26.4 b	6.0 a	3	Cocoa, nutty
885	3-Methylbutanal	21.0 b	22.6 b	27.8 c	20.7 b	6.6 a	0.2	Cocoa, nutty
960	Pentanal	-	21.7 b	21.8 b	24.9 b	20.5 a	12	Nutty, coffee, chocolate
1075	Hexanal	1050.8 c	750.1 b	705.5 b	856.5 b	185.7 a	4.5	Green, grassy
1130	(*E*)-3-Hexenal	1.2 a	1.1 a	1.4 a	1.6 b	1.2 a	160	Green, fruit, apple-like
1135	(*Z*)-3-Hexenal	1.7 a	-	2.3 a	1.6 a	17.9 b	0.25	Green, grassy, leafy
1210	(*E*)-2-Hexenal	52.7 c	24.9 a	36.5 b	36.3 b	24.7 a	17	Green, fruity
1388	Nonanal	91.1 c	113.1 c	105.4 c	75.3 b	7.0 a	1	Green, fatty
1422	(*E*)-2-Octenal	21.2 c	21.2 c	13.6 b	12.8 b	2.0 a	3	Fatty, nutty
1435	(*E*)-6-Nonenal	4.2 b	7.3 c	7.6 c	7.8 c	0.3 a	0.07	Soapy, floral (off-notes)
1445	(*Z*)-6-Nonenal	65.0 c	85.0 d	64.9 c	47.7 b	4.6 a	0.02	Green, cucumber
1499	(*Z*)-2-Nonenal	6.1 b	6.8 b	6.1 b	5.1 b	0.7 a	0.02	Green, cucumber
1531	(*E*)-2-Nonenal	202.9 c	216.3 c	191.3 b	181.8 b	31.8 a	0.08	Green, cucumber
1567	(*E*,*E*)-2,6-Nonadienal	3.0 b	3.4 b	2.9 b	2.0 ab	0.7 a	0.5	Green, cucumber
1581	(*E*,*Z*)-2,6-Nonadienal	243.4 c	226.6 c	185.9 b	182.1 b	59.9 a	0.01	Green, cucumber
1346	Hexanol	101.3 b	129.1 bc	165.9 c	106.8 b	16.4 a	2500	Green, floral
1376	(*Z*)-3-Hexen-1-ol	23.2 b	43.1 c	73.1 d	44.5 c	5.7 a	70	Green, grassy
1656	Nonanol	4.9 b	15.3 c	24.9 d	8.8 b	1.4 a	50	Green, fatty
1680	(*Z*)-3-Nonen-1-ol	134.4 b	247.4 c	274.6 d	221.8 d	5.7 a	-	Fruity, sweet
1709	(*Z*)-6-Nonen-1-ol	6.9 a	22.4 c	24.4 c	12.2 b	0.4 a	1	Green, cucumber
1712	(*E*)-2-Nonen-1-ol	4.3 b	-	-	-	1.9 a	130	Green, fatty
1737	(*E*,*Z*)-3,6-Nonadien-1-ol	0.9 a	3.3 c	4.7 c	1.8 b	0.7 a	3	Fatty, fishy
1747	(*Z*,*Z*)-3,6-Nonadien-1-ol	165.2 b	329.5 c	375.1 d	275.2 c	7.7 a	10	Fatty, fishy
1764	(*E*,*Z*)-2,6-Nonadien-1-ol	12.9 b	31.9 c	27.7 c	15.2 b	1.1 a	0.2	Green, cucumber
1331	6-Methyl-5-hepten-2-one	270.7 c	310.2 c	186.8 b	184.1 b	7.3 a	50	Musty, popcorn, rubber

Different letters (a–d) within each row indicate significant differences of each volatile compound among five samples according to one-way ANOVA and Tukey’s HSD test (α ≤ 0.05). Note: LRI = linear retention index, “-” = not detected. Odor descriptions were mainly adopted from the Good Scents Company. OT: odor threshold in water, was based on L. J. van Gemert’s Compilations of Odour Threshold Values in Air, Water, and Other Media. 2003 and Boelens Aroma Chemical Information Service (BACIS) database.

## Data Availability

Data is contained within the article or Supplementary Materials.

## Supplementary Materials

The following supporting information can be downloaded at: https://www.mdpi.com/article/10.3390/molecules27082536/s1. Table S1: Volatile compounds in watermelon rind, flesh, and rind-flesh blends (peak area %).

## Sample Availability

Samples of the compounds are not available from the authors.
