# Supplementary material for: Free Amino Acids and Volatile Aroma Compounds in Watermelon Rind, Flesh, and Three Rind-Flesh Juices
Source: Molecules. 2022 Apr 14;27(8):2536. doi: 10.3390/molecules27082536 (PMC9027972; doi:10.3390/molecules27082536)
Supplement: Supplementary file 1 [file molecules-27-02536-s001.zip › molecules-1655214-supplementary.pdf]

molecules
MDPI

**Table S1**: Volatile compounds in watermelon rind, flesh, and rind-flesh blends (peak area %).

| V# | LRI | Compound | CAS | 0% Rind | 10% Rind | 20% Rind | 30% Rind | 100% Rind | *p*-value |
|---|---|---|---|---|---|---|---|---|---|
| | | **Aldehydes (32)** | | | | | | | |
| V1 | 672 | Acetaldehyde | 75-07-0 | 4.41 a | 3.87 a | 4.29 a | 4.35 a | 16.95 b | <0.001 |
| V2 | 794 | Propanal | 123-38-6 | 0.97 c | 0.76 b | 0.77 b | 0.94 bc | 0.11 a | <0.001 |
| V3 | 850 | Butanal | 123-72-8 | - | - | 0.09 a | - | 1.19 b | 0.001 |
| V4 | 882 | 2-Methylbutanal | 96-17-3 | 0.39 c | 0.28 a | 0.32 b | 0.31 ab | 0.46 d | <0.001 |
| V5 | 885 | 3-Methylbutanal | 590-86-3 | 0.24 ab | 0.21 a | 0.27 b | 0.24 ab | 0.51 c | <0.001 |
| V6 | 960 | Pentanal | 110-62-3 | - | 0.20 a | 0.21 a | 0.29 a | 1.57 b | <0.001 |
| V7 | 1031 | (*E*)-2-Butenal | 4170-30-3 | 0.11 b | 0.06 a | 0.13 b | - | 2.56 c | <0.001 |
| V8 | 1055 | 2-Ethyl-3-methylbutanal | 26254-92-2 | 0.01 b | 0.005 a | 0.003 a | - | - | 0.011 |
| V9 | 1075 | Hexanal | 66-25-1 | 11.97 bc | 7.05 a | 6.86 a | 9.93 b | 14.16 c | <0.001 |
| V10 | 1121 | (*E*)-2-Pentenal | 1576-87-0 | 0.06 a | - | - | - | 0.13 b | 0.005 |
| V11 | 1130 | (*E*)-3-Hexenal | 69112-21-6 | 0.01 a | 0.01 a | 0.01 a | 0.02 a | 0.09 b | <0.001 |
| V12 | 1135 | (*Z*)-3-Hexenal | 6789-80-6 | 0.02 a | - | 0.02 a | 0.02 a | 1.36 b | 0.005 |
| V13 | 1176 | Heptanal | 111-71-7 | 0.22 b | 0.16 ab | 0.13 a | 0.13 a | - | <0.001 |
| V14 | 1210 | (*E*)-2-Hexenal | 6728-26-3 | 0.69 bc | 0.27 a | 0.41 ab | 0.48 ab | 2.17 c | <0.001 |
| V15 | 1281 | Octanal | 124-13-0 | 0.31 b | 0.26 ab | 0.24 ab | 0.18 a | - | <0.001 |
| V16 | 1315 | (*E*)-2-Heptenal | 57266-86-1 | 0.42 | - | - | - | - | - |
| V17 | 1388 | Nonanal | 124-19-6 | 3.57 c | 3.66 c | 3.53 c | 3.00 b | 1.84 a | <0.001 |
| V18 | 1407 | 2-Methyl-3-methylene-cyclopentane carboxaldehyde | 826337-64-8 | - | - | - | - | 0.27 | - |
| V19 | 1422 | (*E*)-2-Octenal | 2548-87-0 | 0.80 c | 0.66 b | 0.44 a | 0.49 a | 0.51 a | <0.001 |
| V20 | 1435 | (*E*)-6-Nonenal | 2277-20-5 | 0.16 ab | 0.23 bc | 0.25 bc | 0.30 c | 0.08 a | 0.001 |
| V21 | 1445 | (*Z*)-6-Nonenal | 2277-19-2 | 3.34 d | 3.60 e | 2.85 c | 2.49 b | 1.59 a | <0.001 |
| V22 | 1493 | Decanal | 112-31-2 | - | 0.03 a | 0.03 a | 0.02 a | 0.07 a | 0.179 |
| V23 | 1499 | (*Z*)-2-Nonenal | 60784-31-8 | 0.30 a | 0.28 a | 0.26 a | 0.26 a | 0.24 a | 0.742 |
| V24 | 1514 | Benzaldehyde | 100-52-7 | - | 0.09 a | 0.09 a | 0.05 a | 0.52 b | <0.001 |
| V25 | 1531 | (*E*)-2-Nonenal | 18829-56-6 | 10.09 c | 8.87 ab | 8.12 a | 9.20 bc | 10.59 d | <0.001 |
| V26 | 1567 | (*E*,*E*)-2,6-Nonadienal | 17587-33-6 | 0.09 a | 0.08 a | 0.07 a | 0.06 a | 0.14 a | 0.106 |
| V27 | 1581 | (*E*,*Z*)-2,6-Nonadienal | 557-48-2 | 7.08 c | 5.43 b | 4.61 a | 5.38 b | 11.65 d | <0.001 |
| V28 | 1636 | Benzeneacetaldehyde | 122-78-1 | 0.08 b | 0.04 a | 0.04 a | 0.03 a | - | <0.001 |
| V29 | 1637 | (*E*)-2-Decenal | 3913-81-3 | - | 0.03 ab | 0.04 b | 0.02 a | - | 0.034 |
| V30 | 1803 | (*E*,*E*)-2,4-Decadienal | 25152-84-5 | 0.03 a | 0.03 a | 0.01 a | 0.01 a | - | 0.282 |

| | | | | | | | | | |
|---|---|---|---|---|---|---|---|---|---|
| V31 | 1824 | 4-Oxononanal | 74327-29-0 | 0.49 c | 0.27 b | 0.12 a | 0.21 ab | 0.12 a | <0.001 |
| V32 | 2005 | (*E*)-Cinnamaldehyde | 14371-10-9 | 0.01 a | 0.01 a | 0.01 a | 0.01 a | - | 0.027 |
| | | **Alcohols (32)** | | | | | | | |
| V33 | 865 | Methanol | 67-56-1 | 0.45 a | 0.61 b | 0.65 b | 0.85 c | - | <0.001 |
| V34 | 894 | Isopropyl alcohol | 67-63-0 | 0.05 a | 0.05 a | 0.04 a | 0.04 a | 0.23 b | <0.001 |
| V35 | 902 | Ethanol | 64-17-5 | 6.63 d | 4.65 a | 5.53 b | 6.21 c | 6.16 c | <0.001 |
| V36 | 1136 | Butanol | 71-36-3 | 0.07 b | 0.06 ab | 0.08 b | 0.04 a | - | 0.006 |
| V37 | 1149 | 2-Methyl-3-pentanol | 623-37-0 | 0.01 a | 0.01 a | - | - | - | 0.372 |
| V38 | 1152 | 1-Penten-3-ol | 616-25-1 | 0.41 d | 0.33 c | 0.20 a | 0.27 b | 0.90 e | <0.001 |
| V39 | 1199 | 2-Methylbutanol | 137-32-6 | 0.27 ab | 0.25 a | 0.37 b | 0.26 ab | 2.04 c | <0.001 |
| V40 | 1242 | Pentanol | 71-41-0 | 0.60 a | 0.53 a | 0.51 a | 0.50 a | 1.43 b | <0.001 |
| V41 | 1314 | (*Z*)-2-Penten-1-ol | 1576-95-0 | - | 0.30 a | 0.22 a | 0.23 a | 0.39 a | 0.185 |
| V42 | 1346 | Hexanol | 111-27-3 | 3.12 a | 3.28 a | 4.36 b | 3.34 a | 3.37 a | <0.001 |
| V43 | 1356 | (*E*)-3-Hexen-1-ol | 928-97-2 | 0.01 | 0.02 b | 0.02 b | 0.01 a | - | <0.001 |
| V44 | 1376 | (*Z*)-3-Hexen-1-ol | 928-96-1 | 0.29 a | 0.45 b | 0.79 d | 0.57 c | 0.48 bc | <0.001 |
| V45 | 1399 | (*E*)-2-Hexen-1-ol | 928-95-0 | - | - | 0.07 | - | - | - |
| V46 | 1449 | Heptanol | 111-70-6 | 0.04 a | 0.04 a | 0.23 b | 0.20 b | 0.25 b | 0.026 |
| V47 | 1458 | 6-Methylhept-5-en-2-ol | 1569-60-4 | 1.34 b | 0.87 a | 1.41 b | 1.25 ab | - | 0.001 |
| V48 | 1477 | (*Z*)-3-Hepten-1-ol | 2108-05-6 | 0.01 a | 0.05 a | 0.05 b | 0.03 ab | - | 0.011 |
| V49 | 1485 | 2-Ethylhexanol | 104-76-7 | 0.21 a | 0.22 a | 0.26 a | 0.20 a | 0.40 b | 0.001 |
| V50 | 1553 | Octanol | 111-87-5 | 0.38 a | 0.45 a | 0.44 a | 0.29 a | 0.77 b | 0.004 |
| V51 | 1611 | (*E*)-2-Octen-1-ol | 18409-17-1 | 0.40 b | 0.15 a | 0.32 b | 0.10 a | - | 0.023 |
| V52 | 1650 | 2,6-Dimethyl-5-hepten-1-ol | 4234-93-9 | 0.03 a | 0.01 a | 0.02 a | 0.02 | - | 0.511 |
| V53 | 1656 | Nonanol | 143-08-8 | 0.51 a | 1.30 c | 2.18 d | 0.92 b | 1.00 b | <0.001 |
| V54 | 1680 | (*Z*)-3-Nonen-1-ol | 10340-23-5 | 11.22 b | 17.04 c | 19.58 d | 18.84 d | 3.16 a | <0.001 |
| V55 | 1694 | 1-Butynyl-1-cyclopentanol | 155879-96-2 | 0.59 c | 0.26 a | 0.48 b | 0.26 a | 0.67 c | <0.001 |
| V56 | 1709 | (*Z*)-6-Nonen-1-ol | 35854-86-5 | 0.58 a | 1.54 b | 1.74 b | 1.03 ab | 0.20 a | 0.001 |
| V57 | 1712 | (*E*)-2-Nonen-1-ol | 31502-14-4 | 0.26 a | - | - | - | 0.78 b | 0.012 |
| V58 | 1737 | (*E*,*Z*)-3,6-Nonadien-1-ol | 53046-97-2 | 0.03 a | 0.10 ab | 0.15 bc | 0.07 a | 0.18 c | <0.001 |
| V59 | 1747 | (*Z*,*Z*)-3,6-Nonadien-1-ol | 56805-23-3 | 6.08 b | 10.00 c | 11.78 d | 10.30 c | 1.91 a | <0.001 |
| V60 | 1758 | Decanol | 112-30-1 | 0.31 c | 0.06 a | 0.13 b | 0.11 ab | 0.33 c | <0.001 |
| V61 | 1764 | (*E*,*Z*)-2,6-Nonadien-1-ol | 7786-44-9 | 0.47 ab | 0.97 c | 0.87 c | 0.57 b | 0.27 a | <0.001 |
| V62 | 1859 | 3-Ethyl-3-undecanol | 62101-31-9 | 0.47 a | 0.53 a | 0.50 a | 0.55 a | - | 0.560 |
| V63 | 1864 | Benzyl alcohol | 100-51-6 | 0.26 c | 0.19 b | 0.18 b | 0.19 b | 0.04 a | <0.001 |
| V64 | 1895 | Phenylethyl alcohol | 60-12-8 | 0.02 a | 0.02 a | 0.02 a | 0.01 a | - | 0.827 |
| | | **Ketones (18)** | | | | | | | |
| V65 | 983 | 2-Methyl-3-pentanone | 565-69-5 | 0.01 a | 0.01 a | 0.01 a | 0.02 a | - | 0.396 |
| V66 | 1013 | 1-Penten-3-one | 1629-58-9 | 0.17 c | 0.10 b | 0.10 b | 0.12 b | 0.07 a | <0.001 |

| | | | | | | | | | |
|---|---|---|---|---|---|---|---|---|---|
| V67 | 1052 | 2,3-Pentanedione | 600-14-6 | 0.01 b | 0.005 a | - | - | - | 0.015 |
| V68 | 1123 | 4-Methyl-3-penten-2-one | 141-79-7 | 0.08 b | 0.05 a | 0.13 c | 0.13 c | - | 0.009 |
| V69 | 1143 | 3-Heptanone | 106-35-4 | 0.02 a | 0.01 a | - | - | - | 0.570 |
| V70 | 1246 | 3-Octanone | 106-68-3 | 0.30 b | 0.14 a | - | - | - | <0.001 |
| V71 | 1276 | 2-Octanone | 111-13-7 | 0.05 a | 0.03 a | 0.03 a | 0.02 a | - | 0.390 |
| V72 | 1303 | 2,2,6-Trimethylcyclohexanone | 2408-37-9 | 0.02 b | 0.01 a | 0.01 a | 0.01 a | 0.01 a | 0.001 |
| V73 | 1310 | 6-Methyl-6-hepten-2-one | 10408-15-8 | 0.01 a | 0.01 a | 0.01 a | 0.004 a | - | 0.143 |
| V74 | 1320 | 2,5-Octanedione | 3214-41-3 | 0.08 ab | 0.06 a | 0.06 a | 0.09 b | 0.15 c | <0.001 |
| V75 | 1324 | (*Z*)-6-Octen-2-one | 74810-53-0 | 0.03 a | 0.03 a | 0.05 a | 0.04 a | 0.15 b | <0.001 |
| V76 | 1331 | 6-Methyl-5-hepten-2-one | 110-93-0 | 12.40 d | 11.72 d | 7.30 b | 8.57 c | 2.24 a | <0.001 |
| V77 | 1381 | 2-Nonanone | 821-55-6 | 0.04 b | 0.01 a | - | - | - | 0.031 |
| V78 | 1513 | (*E*,*Z*)-3,5-Octadien-2-one | 4173-41-5 | 0.25 c | 0.11 b | 0.05 | 0.05 a | - | <0.001 |
| V79 | 1563 | (*E*,*E*)-3,5-Octadien-2-one | 30086-02-3 | 0.02 a | 0.03 a | - | - | - | 0.644 |
| V80 | 1585 | 6-Methyl-3,5-heptadiene-2-one | 1604-28-0 | 0.06 a | 0.03 a | 0.06 a | 0.04 a | - | 0.751 |
| V81 | 1593 | 2-Undecanone | 112-12-9 | 0.01 a | 0.01 a | 0.01 a | 0.01 a | 0.03 b | 0.048 |
| V82 | 1928 | (Z)-Jasmone | 488-10-8 | 0.02 a | 0.01 a | 0.01 a | 0.01 a | - | 0.168 |
| | | **Tepenes and terpenoids (20)** | | | | | | | |
| V83 | 1146 | β-Myrcene | 123-35-3 | 0.02 a | 0.01 a | 0.02 a | 0.03 a | - | 0.324 |
| V84 | 1192 | Eucalyptol | 470-82-6 | 0.04 a | 0.05 a | 0.04 a | 0.03 a | 0.15 b | 0.005 |
| V85 | 1427 | Tetrahydrolinalool | 78-69-3 | - | 0.003 a | 0.02 ab | 0.01 a | 0.07 b | 0.012 |
| V86 | 1464 | Dihydromyrcenol | 18479-58-8 | 0.04 a | 0.10 a | 0.07 a | 0.05 a | 0.11 a | 0.098 |
| V87 | 1474 | Citronellal | 106-23-0 | 0.004 a | 0.03 b | - | 0.01 a | - | 0.049 |
| V88 | 1545 | Linalool | 78-70-6 | 0.10 a | 0.16 ab | 0.19 ab | 0.12 a | 0.31 b | 0.037 |
| V89 | 1572 | Isobornyl acetate | 125-12-2 | - | 0.02 a | 0.04 b | 0.01 a | 0.10 c | <0.001 |
| V90 | 1633 | Menthol | 15356-70-4 | 0.02 a | 0.02 a | 0.01 a | 0.03 a | 0.05 b | 0.001 |
| V91 | 1674 | *cis*-Citral | 106-26-3 | 0.33 c | 0.41 d | 0.21 b | 0.22 b | 0.08 a | <0.001 |
| V92 | 1725 | *trans*-Citral | 141-27-5 | 0.70 b | 0.89 c | 0.68 b | 0.62 b | 0.18 a | <0.001 |
| V93 | 1795 | *cis*-Geraniol | 106-25-2 | 0.03 a | 0.02 a | 0.01 a | 0.01 a | - | 0.223 |
| V94 | 1839 | *trans*-Geraniol | 106-24-1 | 0.08 a | 0.08 a | 0.07 a | 0.09 a | - | 0.133 |
| V95 | 1846 | *trans*-Geranylacetone | 3796-70-1 | 1.44 d | 1.24 c | 0.99 b | 0.97 b | 0.31 a | <0.001 |
| V96 | 1917 | (*E*)-β-Ionone | 79-77-6 | 0.07 a | 0.08 a | 0.06 a | 0.06 a | 0.07 a | 0.731 |
| V97 | 1938 | 6,10-Dimethyl-5,9-undecadien-2-ol | 53837-34-6 | 0.01 a | 0.01 a | 0.01 a | 0.01 a | - | 0.393 |
| V98 | 1964 | β-Ionone-5,6-epoxide | 23267-57-4 | 0.02 a | 0.02 a | 0.01 a | 0.01 a | - | 0.095 |
| V99 | 2010 | (*E*,*Z*)-Psuedoionone | 13927-47-4 | 0.01 a | 0.01 a | 0.01 a | 0.01 a | - | 0.930 |
| V100 | 2087 | (*E*,*E*)-Psuedoionone | 3548-78-5 | 0.02 b | 0.02 b | 0.01 a | 0.01 a | - | 0.002 |
| V101 | 2241 | Dihydroactinidiolide | 15356-74-8 | 0.01 b | 0.005 a | 0.004 a | 0.01 b | - | 0.009 |
| V102 | 2273 | Farnesyl acetone | 1117-52-8 | 0.01 a | 0.01 a | 0.01 a | 0.01 a | - | 0.484 |
| | | **Esters and lactones (9)** | | | | | | | |

| | | | | | | | | | |
|---|---|---|---|---|---|---|---|---|---|
| V103 | 858 | Ethyl acetate | 141-78-6 | 0.10 ab | - | 0.04 a | - | 0.18 b | <0.001 |
| V104 | 1165 | Pentyl acetate | 628-63-7 | 0.01 a | 0.01 a | 0.01 a | 0.01 a | - | 0.194 |
| V105 | 1265 | Hexyl acetate | 142-92-7 | 0.01 a | 0.01 a | 0.01 a | 0.01 a | - | 0.052 |
| V106 | 1601 | (*Z*)-3-Nonenyl acetate | 13049-88-2 | - | - | 0.02 a | 0.02 a | - | 0.485 |
| V107 | 2020 | Methyl 9-oxo-nonanoate | 1931-63-1 | 0.01 a | 0.01 a | 0.005 a | 0.01 a | - | 0.711 |
| V108 | 2156 | Methyl hexadecanoate | 112-39-0 | 0.004 a | 0.01 a | 0.01 a | 0.01 a | 0.03 b | 0.001 |
| V109 | 2362 | Methyl (*Z,Z*)-9,12-octadecadienoate | 112-63-0 | - | - | 0.004 a | 0.005 a | 0.02 b | 0.002 |
| V110 | 1996 | γ-Nonalactone | 104-61-0 | 0.04 a | 0.03 a | 0.03 a | 0.03 a | 0.08 b | <0.001 |
| V111 | 2103 | *cis*-Jasmone lactone | 70851-61-5 | 0.01 a | 0.002 a | 0.003 a | 0.01 a | - | 0.329 |
| | | **Acids (5)** | | | | | | | |
| V112 | 1851 | Hexanoic acid | 142-62-1 | 0.13 a | 0.10 a | 0.14 a | 0.15 a | - | 0.449 |
| V113 | 1942 | 2-Ethylhexanoic acid | 149-57-5 | 0.01 a | 0.01 a | 0.02 a | 0.01 a | - | 0.732 |
| V114 | 1947 | Heptanoic acid | 112-05-0 | 0.03 a | 0.03 a | 0.04 a | 0.04 a | 0.05 a | 0.608 |
| V115 | 2039 | Octanoic acid | 124-07-2 | 0.02 a | 0.02 a | 0.04 a | 0.04 a | 0.02 a | 0.158 |
| V116 | 2127 | Nonanoic acid | 112-05-0 | 0.01 a | 0.01 a | 0.02 ab | 0.03 ab | 0.04 b | 0.044 |
| | | **Sulfides (3)** | | | | | | | |
| V117 | 648 | Methanethiol | 74-93-1 | 0.50 a | 0.46 a | 0.39 a | 0.53 a | 0.18 a | 0.157 |
| V118 | 1061 | Dimethyl disulfide | 624-92-0 | 0.02 a | 0.02 a | 0.02 a | 0.02 a | 0.32 b | <0.001 |
| V119 | 1367 | Dimethyl trisulfide | 3658-80-8 | - | - | - | 0.01 a | 0.17 b | 0.001 |
| | | **Others (7)** | | | | | | | |
| V120 | 928 | 2-Ethylfuran | 3208-16-0 | 0.02 a | 0.02 b | 0.01 a | 0.03 b | 0.01 a | <0.001 |
| V121 | 1223 | 2-Pentylfuran | 3777-69-3 | 1.07 bc | 2.11 d | 1.05 b | 1.40 c | 0.55 a | <0.001 |
| V122 | 1294 | (*E*)-2-pentenylfuran | 70424-14-5 | 0.42 c | 0.66 d | 0.28 b | 0.47 c | 0.16 a | <0.001 |
| V123 | 1413 | 3-(4-Methyl-3-pentenyl)-furan | 539-52-6 | 0.09 bc | 0.11 c | 0.08 abc | 0.06 ab | 0.04 a | 0.001 |
| V124 | 1113 | Ethylbenzene | 100-41-4 | 0.02 a | 0.02 ab | 0.02 ab | 0.01 a | 0.03 b | 0.017 |
| V125 | 1170 | o-Xylene | 95-47-6 | 0.03 ab | 0.02 a | 0.05 b | 0.03 ab | - | 0.001 |
| V126 | 1728 | Naphthalene | 91-20-3 | - | - | - | - | 0.22 | - |

Different letters within each row indicate significant differences of each volatile compound among five samples according to one-way ANOVA and Tukey's HSD test ($\alpha \leq 0.05$). LRI = linear retention index; "-" = not detected.
